# The role of bone marrow-derived cells in the origin of liver cancer revealed by single-cell sequencing

**DOI:** 10.20892/j.issn.2095-3941.2019.0369

**Published:** 2020-02-15

**Authors:** Lu Chen, Xianfu Yi, Piao Guo, Hua Guo, Ziye Chen, Chunyu Hou, Lisha Qi, Yongrong Wang, Chengwen Li, Peng Liu, Yucun Liu, Yuanfu Xu, Ning Zhang

**Affiliations:** ^1^The concrete information of affiliations should be presented, for instance, Department of Tianjin Medical University Cancer Institute and Hospital, National Clinical Research Center for Cancer, Key Laboratory of Cancer Prevention and Therapy, Tianjin’s Clinical Research Center for Cancer, Tianjin 300070, China; ^2^School of Biomedical Engineering and Technology, Tianjin Medical University, Tianjin 300070, China; ^3^The Center for Translational Cancer Research, Peking University First Hospital, Beijing 100034, China; ^4^State Key Laboratory of Experimental Hematology, National Clinical Research Center for Blood Diseases, Institute of Hematology & Blood Diseases Hospital, Chinese Academy of Medical Sciences & Peking Union Medical College, Tianjin 300020, China; ^5^CAMS Key Laboratory for Prevention and Control of Hematological Disease Treatment Related Infection, Tianjin 300020, China; ^6^Cytogenetics Laboratory, Institute of Hematology and Blood Diseases Hospital, Chinese Academy of Medical Sciences and Peking Union Medical College, Tianjin 300020, China

**Keywords:** Hepatocellular carcinoma, bone marrow-derived cells (BMDCs), origination, genome sequencing, copy number alteration

## Abstract

**Objective:** Epithelial cancers often originate from progenitor cells, while the origin of hepatocellular carcinoma (HCC) is still controversial. HCC, one of the deadliest cancers, is closely linked with liver injuries and chronic inflammation, which trigger massive infiltration of bone marrow-derived cells (BMDCs) during liver repair.

**Methods:** To address the possible roles of BMDCs in HCC origination, we established a diethylnitrosamine (DEN)-induced HCC model in bone marrow transplanted mice. Immunohistochemistry and frozen tissue immunofluorescence were used to verify DEN-induced HCC in the pathology of the disease. The cellular origin of DEN-induced HCC was further studied by single cell sequencing, single-cell nested PCR, and immunofluorescence-fluorescence *in situ* hybridization.

**Results:** Studies by using single cell sequencing and biochemical analysis revealed that HCC cells in these mice were coming from donor mice BMDCs, and not from recipient mice. Furthermore, the copy numbers of mouse orthologs of several HCC-related genes previously reported in human HCC were also altered in our mouse model. DEN-induced HCCs exhibited a similar histological phenotype and genomic profile as human HCCs.

**Conclusions:** These results suggested that BMDCs are an important origin of HCC, which provide important clues to HCC prevention, detection, and treatments.

## Introduction

The identification of cancer cell origin provides important information for cancer pathogenesis, early detection, and treatment. Tissue stem/progenitor cells play an important role in the origination of several cancers, including intestinal cancer^1^, melanoma^2^, and breast cancer^3^. However, the origin of liver cancer is still under investigation. Prior studies have demonstrated that oval cells are associated with hepatoma *via* activation of the Hippo pathway^4^. Lineage-tracing studies have revealed that hepatocellular carcinoma (HCC) does not originate from the progenitor/biliary compartment but from mature hepatocytes^5^. The high plasticity of mature hepatocytes further complicates the investigation^6^. However, the origins of HCC are still controversial.

One key feature of HCC is its close association with liver injuries and chronic inflammation, which induces bone marrow-derived cell (BMDC) infiltration for liver repair^7^. Several reports have suggested that BMDCs are the origins of a number of epithelial cancers including gastric cancer^8^, basal cell carcinoma^9^, and lung adenocarcinoma^10^. In the present study, we investigated the role of BMDCs in HCC origination by using a bone marrow transplant HCC mouse model.

## Materials and methods

### Animals

Male and female wild-type C57BL/6 mice were purchased from Nanjing Biomedical Research Institute of Nanjing University (Nanjing, Jiangsu, China). Male C57BL/6 transgenic mice expressing enhanced green fluorescent protein (GFP) (GFP transgenic mice) were developed at the Institute of Hematology and Blood Diseases Hospital, Chinese Academy of Medical Sciences and Peking Union Medical College (Tianjin, China). Mice were housed in colony cages with a 12 h light/dark cycle. This study was approved by the Ethics Committee of the Tianjin Medical University Cancer Institute and Hospital, China (Approval No. 2012094).

### Bone marrow transplantation

Six-week-old GFP transgenic mice (male) were used as the bone marrow donor mice. Total bone marrow was flushed from the marrow cavity of the femurs and tibias using a 1 mL syringe, and successively passed through a 70 µm and 40 µm nylon mesh cell strainer (Corning, Corning, NY, USA) to produce a single cell suspension in phosphate-buffered saline (PBS). Then, the red blood cells were excluded using an ammonium chloride-potassium (ACK) lysis buffer. Recipient wild-type C57BL/6 mice (male) were irradiated twice with a dose of 4.5 Gys per time, from an X-ray irradiator. The interval was 4 h. A total of 1 × 10^6^ donor marrow cells were then injected once into the tail vein of these mice. After 4 weeks of recovery, these mice were used for further experiments.

### Animal model of HCC

To induce HCC, the genotoxic carcinogen diethylnitrosamine (DEN; Sigma-Aldrich, St. Louis, MO, USA) was administered in drinking water for 16 weeks at a concentration of 30 mg/L. Then, the mice were sacrificed, and the tumor identity of the liver neoplasm was confirmed by hematoxylin and eosin (H&E) staining.

### Immunohistochemistry

Paraffin-embedded liver sections were deparaffinized and rehydrated with xylene and graded concentrations of ethanol. After microwave antigen retrieval and endogenous peroxidase activity blocking, primary antibodies against alpha fetoprotein (AFP, 14550-1-AP; Proteintech, Rosemont, IL, USA), CD34 (ab81289), cytokeratin 19 (CK19, ab52625), and glypican 3 (GPC3, ab66596) (Abcam, Cambridge, UK), and the secondary anti-rabbit antibody (PV-6002; Zhongshan Golden Bridge Biotechnology, Beijing, China) were used to incubate the tissue sections. Immunostaining was detected using 3,3′-diaminobenzidine staining and counterstaining with 10% Mayer hematoxylin.

### Frozen tissue immunofluorescence

The tissue slices that were cryopreserved at −80 °C were recovered at room temperature for 5 min. Then, these slices were fixed in ice acetone for 10 min. After being washed with PBS and blocked in 3% bovine serum albumin (BSA), the slices were incubated with primary antibodies against GPC3 (Abcam) and GFP (ab6673; Abcam) overnight at 4 °C and Alexa Fluor 488- and 594- conjugated secondary antibodies (Life Technologies, Carlsbad, CA, USA) at 37 °C for 1 h. Cell nuclei were counterstained with diamidinophenylindole (DAPI) (Solarbio, Beijing, China). All images were captured with a confocal laser scanning microscope (Carl Zeiss, Oberkochen, Germany).

### Single cell acquisition

The isolated liver tumor was minced and incubated with the digestion solution (Tumor Dissociation Kit; Miltenyi Biotec, Bergisch Gladbach, Germany) at 37 °C for 1 h. A single cell suspension was generated by successively passing the digestion product through 70 µm and 40 µm nylon mesh cell strainers (Corning). Then, the ACK lysis buffer was applied to exclude red blood cells, and CD45 microbeads (Miltenyi Biotec) were used to remove leukocytes. Single cells were pipetted into individual polymerase chain reaction (PCR) tubes and analyzed under a fluorescence microscope (Leica, Wetzlar, Germany).

### Whole genome amplification, library preparation, and sequencing

The whole genome of each single cell was amplified using a method called “multiple annealing and looping-based amplification cycles” (MALBAC)^11^. The genomic integrity of the amplification products was evaluated using quantitative PCR, which was performed on 8 randomly selected loci, each on a different chromosome (**Supplementary Table S1**). At least 7 of these 8 loci amplifying with a reasonable cycle threshold (Ct) number (23–26) was the criterion of cells suitable for further sequencing studies. The amplified DNA products were then used to construct sequencing libraries with the NEBNext, DNA Library Prep Master Mix Set for Illumina (New England Biolabs, Ipswich, MA, USA) according to the manufacturer’s protocol. After a quality check, the libraries were sequenced on an Illumina HiSeq X Ten system (San Diego, CA, USA) (read lengths of 2 × 150 bp).

### Single cell sequencing data analysis

Single cell sequencing data were analyzed using methods described previously^11^ with minor modifications. Clean sequence reads stored in FASTQ format were mapped to mouse genome assembly mm10/GRCm38, using Bowtie 2 with default parameters. Samtools (1.3.1) was used to sort BAM files, eliminate PCR duplicates, and filter out reads with low mapping quality (MQ < 40). The aligned data were counted in fixed width bins of 500 kb, while aberrant bins were filtered out. After Loess normalization for GC-content bias, copy number profiles were segmented using the Circular Binary Segmentation method with parameters of alpha = 0.01 and undo. prune = 0.05. Then, Merge Levels was used to merge adjacent segments with non-significant differences. To construct the event matrix, a multiple-sample population segmentation algorithm was applied to each mouse single cell sample using a copy number with penalty parameter gamma = 20. Then, the segments smaller than 10 bins were removed, and the event state was calculated with cutoffs of copy number ratios of 1.4 and 0.6 for amplification and deletion, respectively. Copy number ratios less than 1.3 and greater than 0.7, which meant that the copy numbers were between 2.6 and 1.4, were classified as neutral diploid. Other ambiguous ratios were classified to the most likely states using Bayesian inference. Based on event matrices ignoring events on sex chromosomes, maximum parsimony trees were built using the parsimony ratchet algorithm. The trees were rooted by the normal diploid blood cells. Finally, he ACCTRAN criterion was used to infer branch lengths and ancestral character probability distributions.

### Single cell nested PCR of GFP sequences

The detection of the GFP sequence in a single cell genome was conducted with 3 sets of oligonucleotide primers (**Supplementary Table S2**). Primers 1, 3, and 5 were the outer primers, and primers 2, 4, and 6 were the inner primers. Two rounds of PCR amplification were performed with OneTaq Hot Start Quick-load Master Mix with Standard Buffer (New England Biolabs) in a DNA thermal cycler (BioRad, Hercules, CA, USA). The outer primers were used in the first PCR. The thermal cycling conditions were 90 s at 94 °C, followed by 15 cycles of 30 s at 94 °C, 45 s at 59 °C/60 °C, 30 s at 68 °C, and 5 min at 68 °C for the final extension. The PCR product was then used as the template for the second reaction, where the inner primers were also included. The PCR protocol was the same as the first PCR, except that the cycle number was 30. The PCR products were electrophoresed using a 2% agarose gel, and the staining was visualized with 0.25 µg/mL ethidium bromide (Solarbio) on an ultraviolet transilluminator (BioRad).

### Flow cytometry

For the evaluation of bone marrow transplantation efficiency, 20 µL of fresh blood from a recipient mouse tail was lysed using ACK lysis buffer and washed once in PBS. Then, the cell pellet was resuspended in staining buffer (0.5% BSA and 2 mM EDTA in PBS) and stained with anti-CD45-PE antibody (130–102-596; Miltenyi Biotec) according to the manufacturer’s protocol. The isotype control antibody was Rat IgG2b (130-102-663; Miltenyi Biotec). The percentage of GFP-positive cells was analyzed after the CD45^+^ cell population was sorted using a flow cytometer.

### Immunofluorescence-fluorescence *in situ* hybridization

Single cell preparations from liver tumors of sex-mismatched and bone marrow-transplanted mice were prepared as detailed above. An aliquot of cells (~9,000 cells) was spun onto slides at a speed of 700 rpm for 5 min. The slides were then fixed in 100% methanol overnight at room temperature. After methanol was completely evaporated, the cells were permeabilized with 0.2% Triton X-100 in PBS. The slides were then incubated with the primary antibody for GPC3 (Abcam) overnight at 4 °C, followed by incubation with the Alexa Fluor 594-conjugated secondary antibody (Life Technologies) for 1 h at 37 °C. Cell nuclei were counterstained with 4′,6-diamidino-2-phenylindole (DAPI) (Cytocell, Cambridge, UK). Cytoplasmic staining of GPC3 was visualized using a fluorescence microscope (Carl Zeiss), and the location of cells was recorded. Next, the slides were incubated with 0.1 mg/mL pepsin (Sigma-Aldrich) for 5 min at 37 °C. The slides were then washed with 2× Saline Sodium Citrate (SSC), dehydrated with an ethanol series, and air-dried. The red-labeled chromosome X probe and green-labeled chromosome Y probe (ID Labs, London, Canada) were mixed and applied onto the slides. The probes and chromosomal DNAs were hybridized for 18 h at 37 °C, following co-denaturation for 2 min at 69 °C. After a post-hybridization wash, the slides were counterstained with DAPI for nuclear staining. The fluorescence images of chromosomes X and Y from cells whose locations had been recorded previously were obtained with a fluorescence microscope (Carl Zeiss).

### Statistical analysis

All statistical analyses were performed using SPSS statistical software for Windows, version 25.0 (SPSS, Chicago, IL, USA). Error bars represent the standard error of the mean (SEM). The analyses were performed using the unpaired two-tailed Student’s *t-*test. A value of *P* < 0.05 was considered statistically significant.

### Data availability

The datasets generated and/or analyzed during the current study are available from the corresponding author on reasonable request.

## Results

### Histopathology revealed the potential origin of HCCs

**Figure 1:**
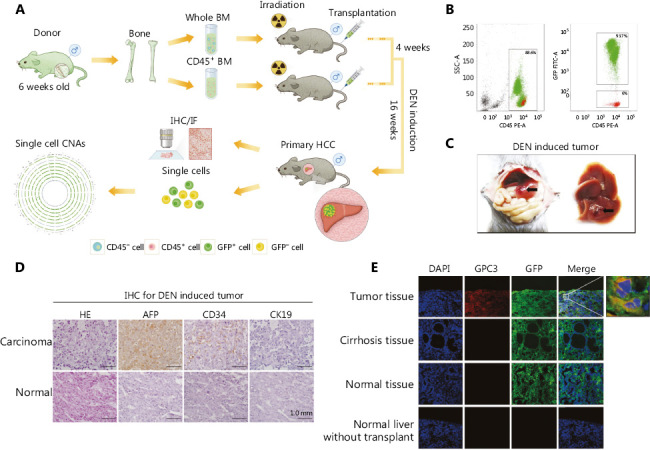
Histopathology revealed the potential origin of hepatocellular carcinoma (HCC). (A) Schematic diagram of the experimental design. (B) The efficiency of bone marrow transplantation was assessed by analysis of green fluorescent protein (GFP) in peripheral leukocytes using flow cytometry. (C) Representative pictures of the mouse liver. Arrows indicate hepatocarcinogenic nodules. (D) Diethylnitrosamine (DEN)-induced HCC was verified by hematoxylin and eosin (H&E) staining and immunohistochemistry, scale bar = 1.0 mm. (E) Colocalization of GFP and glypican 3 (GPC3) in the experimental group (DEN induction group-tumor tissue and cirrhosis tissue) and the control group (normal liver without DEN induction and normal liver without transplantation), as determined by means of immunofluorescence.

To investigate the contribution of BMDCs in the origination of HCC, we established an allogeneic bone marrow transplant C57BL/6 mouse model that was further induced to develop HCC by diethylnitrosamine (DEN) treatment (**Figure 1A**). The following three groups were established as controls: DEN-induced wild-type mice (without bone marrow transplantation), bone marrow-transplanted mice without DEN treatment, and wild-type mice. In detail, after lethal irradiation, 6-week-old wild-type mice were transplanted with GFP labeled whole bone marrow cells and 4 weeks later DEN was added to their drinking water to further induce HCC for another 16 weeks (**Figure 1A**).

After 4-week transplantation, flow cytometry analysis showed that GFP^+^ cells accounted for 93% of mouse peripheral leukocytes, indicating the success of bone marrow transplantation (**Figure 1B**). After 16 weeks of DEN treatment, 3 mice developed detectable nodules in their livers (1 mouse died), while the other 3 mice showed inflamed livers without apparent nodules in the bone marrow-transplanted group (**Figure 1C, Supplementary Table S3**). In the control groups, 16 weeks of treatment with DEN induced tumors in 3 out of 6 wild-type mice. Bone marrow transplantation alone did not induce any tumor formation.

DEN-induced liver nodules were examined by pathological analysis. H&E staining of nodules showed typical HCC morphology (**Figure 1D**). Consistently, a strong alpha fetoprotein (AFP) signal was detected in the tumor tissue. Additionally, CD34 staining was also positive in tumor nodules, indicating angiogenesis in the tumor tissue, which is a common feature in HCC. However, staining for cytokeratin 19 (CK19), a biomarker for cholangiocarcinoma, was negative. We further confirmed the HCC development by staining of glypican 3 (GPC3) present in the frozen tumor tissue but not expressed in the cirrhosis and normal liver tissues (**Figure 1E**).

More importantly, large numbers of cells in the tumor tissue showed co-staining for GPC3 and GFP, suggesting that these HCC cells originated from BMDCs of donor mice, but not the recipient mouse livers (**Figure 1E**). GFP signals were also detected in the livers of bone marrow-transplanted mice without tumors, likely from infiltrated blood cells. Taken together, these results indicated that donor bone marrow cells might be a potential origin of HCC cells.

### Single-cell sequencing revealed the origination and evolution of HCC in the whole bone marrow transplantation mice

**Figure 2:**
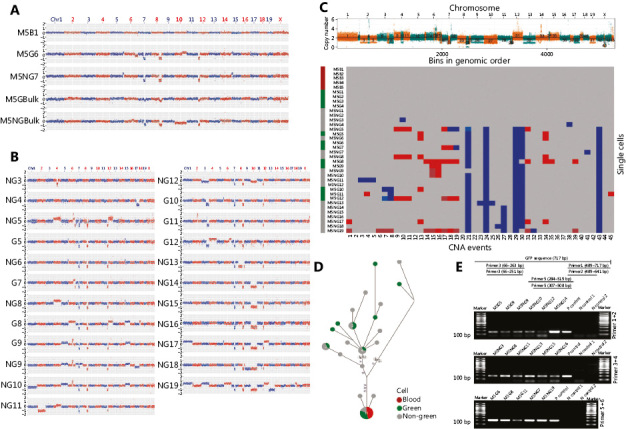
Single-cell sequencing revealed the origination and evolution of hepatocellular carcinoma (HCC) in the whole bone marrow transplantation mice. (A) Representative copy number alteration (CNA) patterns of single tumor cells from diethylnitrosamine (DEN)-induced HCC in Mouse #5. The copy number profile (blue and red dots) indicated by log2 (copy number ratio) is plotted along the genome with a bin size of 500 kb. (M5: Mouse #5, B: blood cell, G: green cell, NG: non-green cell, Bulk: include 100 cells). (B) CNA patterns of single tumor cells from DEN-induced HCC in Mouse #5. The copy number profile indicated by log2 (copy number ratio) is plotted along the genome with a bin size of 500 kb. (Green: green cell, gray: non-green cell). (C) Multiple-cell segmentation (top) and trinary event matrices (bottom) for HCC from whole bone marrow transplantation mice. (D) A phylogenetic tree was constructed based on the CNA trinary event matrices of single cells. (E) Nested polymerase chain reaction (PCR) was used for detecting GFP at the single-cell level (P control: positive control from the genomic DNA of GFP mouse liver tissue. N control: negative control from wild-type mouse peripheral lymphocytes).

Somatic copy number alteration (CNA), a major signature of cancer cells, was used to distinguish tumor cells from non-tumor cells in these nodules at single-cell levels^12,13^. In Mouse #5 (**Supplementary Table S3**), after the removal of lymphocytes using CD45 microbeads in the cell suspension from tumor nodules, both green and non-green cells (GCs and NGCs, respectively) were collected. Then, each single cell was amplified by multiple annealing and looping-based amplification cycles (MALBAC) and assessed by low-depth (0.3×) whole-genome sequencing (**Figure 2A**). We determined that many CNA-positive cells were green cells, indicating that these tumor cells originated from donor bone marrow.

To our surprise, many non-green cells also showed CNAs. There were two possible explanations for this result: either these non-green cells originated from recipient mice therefore from a different origin, or they were from the same origin from donor bone marrow but lost their GFP expression during tumorigenesis. The CNA pattern similarities between green and non-green cells, for examples, indicated CNAs at chromosomes 7, 8, and 12, suggesting that they were from the same origin (**Figure 2A and 2B**). Next, copy number segmentation was achieved by using a multiple-sample breakpoint algorithm to identify common chromosome breakpoints that occurred across single cells among green and non-green cells (**Figure 2C**). Phylogenetic tree analyses of single-cell CNA events further indicated that most cancer green and non-green cells were originated from one single origin (**Figure 2D**).

Given the above findings, we speculated that the green and non-green tumor cells likely shared the same origin, namely the donor bone marrow, while tumor non-green cells lost their GFP expression during tumor progression. To further test this hypothesis, we performed nested PCR on GFP single-cell levels (**Figure 2E, Supplementary Table S2**). Here, 62.5% of tumor green cells and 70.6% of tumor non-green cells showed positive GFP PCR signals (**Supplementary Table S4**). The loss of positive PCR signals in some tumor cells was probably due to the coverage limitation of the MALBAC technique^14^. PCR results further indicated that tumor non-green cells contained the *GFP* gene and were also from donor mouse bone marrow. Taken together, both the phylogenetic evolution analyses and nested PCR assays for GFP indicated that the green and non-green tumor cells shared a common origin, coming from the donor BMDCs.

### Validation of the origination and evolution of DEN-induced HCC in mice as revealed by single-cell sequencing

**Figure 3:**
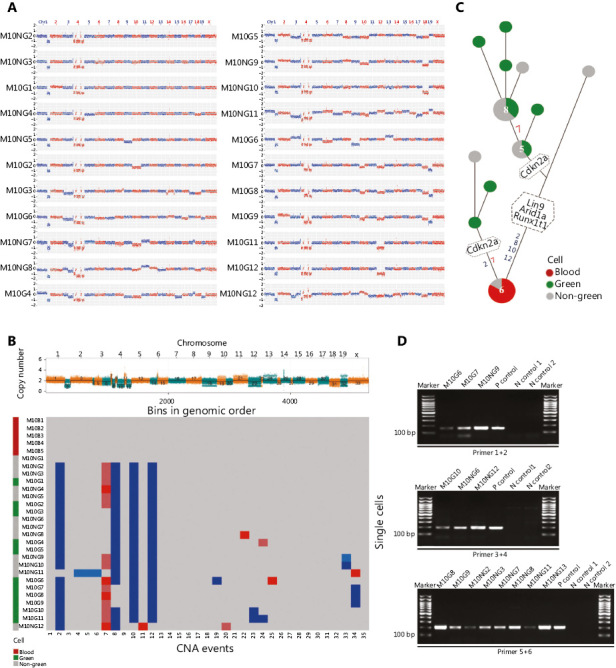
Following validation of the origination and evolution of DEN-induced HCC in mice as revealed by single-cell sequencing. (A) CNA patterns of single tumor cells from a DEN-induced HCC in whole bone marrow transplantation mice (Mouse #10). The copy number profile indicated by log2 (copy number ratio) is plotted along the genome with a bin size of 500 kb. (Green: green cell, gray: non-green cell). (B) Multiple-cell segmentation (top) and trinary event matrices (bottom) for HCC in Mouse #10. (C) The phylogenetic tree was constructed based on the CNA trinary event matrices of single cells of HCC in Mouse #10. (D) Nested PCR for detecting GFP at the single-cell level in Mouse #10. P control: positive control from the genomic DNA of GFP mouse liver tissue. N control: negative control from wild-type mouse peripheral lymphocytes. Abbreviation are defined in **Figures 1 and 2**.

For further confirmation, DEN induced HCC in 8 out of 25 (32%) mice (**Supplementary Table S5**; Mouse #10, #14, #17, #22, #24, #25, #29, and #30) in the validation group (**Supplementary Table S5**). Among the 8 tumor mice, 3 mice were used for histopathology examinations (**Supplementary Table S5**; Mouse #14, #22, and #30). GPC3 signals appeared to be colocalized with GFP signals, confirming that many HCC cells were from donor bone marrow (**Supplementary Figure S1**). Tumors from the other 5 mice (Mouse #10, #17, #24, #25, and #29) were used for single-cell sequencing, phylogenetic evolution tree analyses, and nested PCR for GFP. As shown in **Figure 3**, **Supplementary Figure S2-S7**, tumor cells from each mouse, as evidenced by shared CNAs, appeared to be mainly evolved from one single origin from donor bone marrow (**Figure 3A, Supplementary Figure S2, S3, and S4**). Phylogenetic evolution analyses revealed that most tumor cells from 1 mouse were of a single origin (**Figure 3B and 3C, Supplementary Figure S5, S6, and S7**). Nested PCR showed positive GFP bands in 81.8% (Mouse #10), 80% (Mouse #17), 57.1% (Mouse #24), and 75% (Mouse #25) of tumor non-green cells (**Supplementary Table S4, Figure 3D, Supplementary Figure S5, S6 and S7**). Thus, HCC tumors from these mice also originated from donor bone marrow.

Mouse #29 appeared to be at an early stage of HCC tumorigenesis. Only 1–2 CNAs were detected in each single cell; therefore, the data could not be used to construct an evolution tree (**Supplementary Figure S8 and S9**). Most CNA-positive cells from bone marrow were green. Nested PCR results showed that 1 tumor NGC (M29NG10) contained a GFP sequence and therefore must have also been from donor bone marrow (**Supplementary Figure S9C**). The other tumor NGC (M29NG11) was GFP-negative as shown by nested PCR. Thus, the tumor cells from M29 mostly originated from donor bone marrow cells. In summary, all the examined mice that received whole bone marrow transplants appeared to develop HCC from donor bone marrow.

### HCC cells from male mice transplanted with female mouse bone marrow contained only X chromosomes, without Y chromosomes

**Figure 4:**
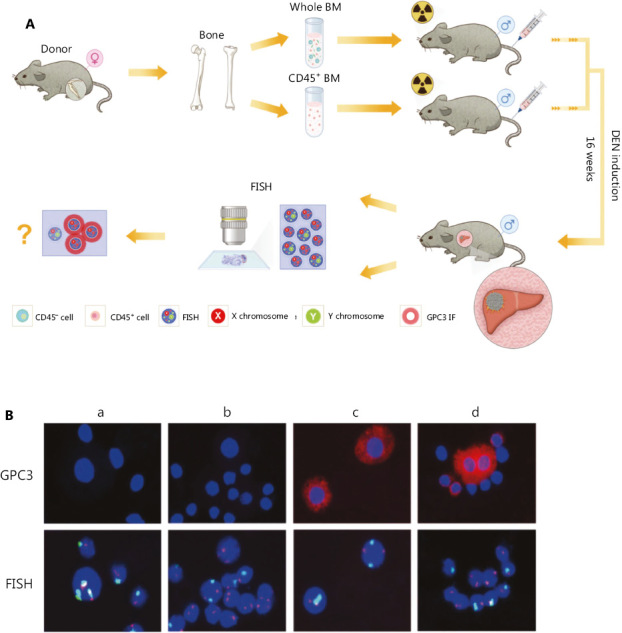
HCC cells from male mice transplanted with female mouse bone marrow contained only X chromosomes, without Y chromosomes. (A) Construction of the experimental animal model and schematic diagram of the FISH process. (B) X (red) and Y (green) fluorescence *in situ* hybridization (FISH) confirmed that all GPC3^+^ HCC cells originated from bone marrow (Group a: nontransplantation, non-DEN induction; Group b: Bone marrow (transplantation, non-DEN induction; Group c: nontransplantation, DEN induction; Group d: whole bone marrow transplantation, DEN induction). Abbreviations are defined in **Figures 1 and 2**.

Mouse hepatocytes are polyploidy, which may be partially due to cell fusion^15^. To further examine the possibility that the tumor cells may originate from the fusion between normal donor bone marrow cells and recipient liver tumor cells, we transplanted bone marrow from female mice to irradiated male mice, followed by 16 weeks of DEN induction, as illustrated in **Figure 4A**. The tumors were dissociated into single-cell suspensions for a combined analysis of sex chromosomes and GPC3 expression. We examined 100 GPC3-positive cells from each tumor in the whole bone marrow-transplanted mice and found that these cancer cells did not contain Y chromosomes, indicating that these tumor cells were originated from the female donor bone marrow (**Figure 4B**). Therefore, the experiment further confirmed that BMDCs play an important role in HCC origination.

### The DEN-induced HCC model appeared to be highly relevant to human HCC

**Figure 5:**
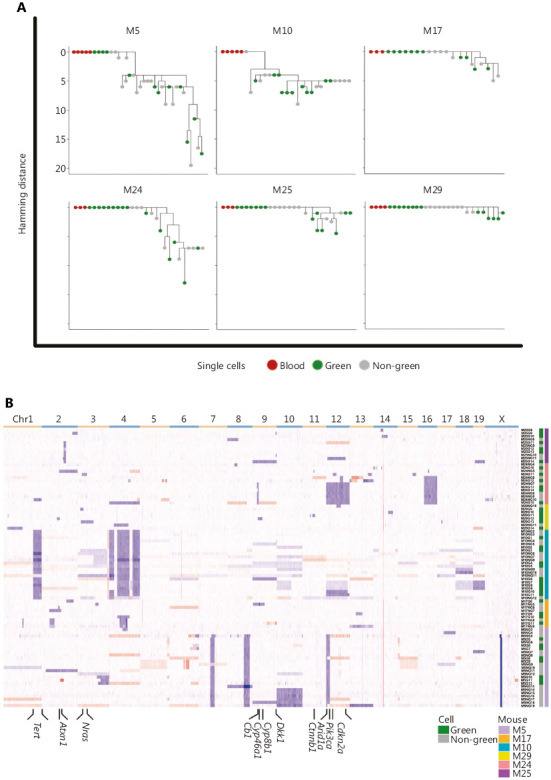
The DEN-induced HCC model appeared to be highly relevant to human HCC. (A) Maximum-parsimony trees for HCC tumors from 6 mice. (B) Heatmap of CNAs of all 88 tumor cells in the 6 HCC mice. The color from blue to red indicates values in the form of log2 (copy number ratio) from −2 to 2. Abbreviations defined in **Figures 1 and 2**.

Recent work by Gao et al.^16^ clearly indicated that the CNA evolution of triple-negative breast cancer cells followed a punctuated burst model, not a gradual evolution model. This observation shed new light on tumor initiation and progression, and has been confirmed in colorectal cancer as well^17^. The same punctuated burst model was clearly presented in our DEN-induced mouse HCC model, as shown in Mouse #5 and Mouse #10 (**Figure 5A**). However, we did detect mouse HCC at a very early stage in Mouse #29, with only 1-2 CNAs, suggesting that a punctuated burst was not essential for HCC development. The CNA results from Mouse #5 and Mouse #24 also appeared to support the gradual evolutional model (**Figure 5A**). Therefore, our results suggested that both gradual and punctuated burst models possibly mediated HCC initiation and progression in mice. The exact CNA evolution models in DEN-induced mouse HCCs will need more studies and detailed interpretation.

Multiple transgenic and chemically-induced mouse models have been developed to investigate various aspects of HCC^18^. A comprehensive genetic analysis showed that the DEN-induced mouse HCCs closely resembled those of the poorer survival group of human HCCs^19^. In our investigation, DEN-induced HCCs exhibited a similar histological phenotype as human HCCs (**Figure 1D**). Next, we clustered the single-cell sequencing data from all the mice (**Figure 5B**). Bioinformatic analyses showed that single cells from individual mice with HCC were genetically more related to each other than to cells from other mice, based on the CNA profiles, suggesting that these cells shared a common ancestral lineage. Furthermore, the copy numbers of mouse orthologs of several HCC-related genes previously reported in human HCC^20^ were also altered in our mouse HCC model (**Figure 5B, Supplementary Table S6**). Therefore, our DEN-induced HCC model appeared to be highly relevant to human HCCs.

## Discussion

There has been a heated debate on the cellular origin of HCC. A number of previous studies have proven the potential origin of HCC by using different models. Multiple cell types resident in the liver might initiate tumor growth, such as hepatocytes, biliary epithelial cells, and adult stem/progenitor cells. Among these cell types, the notion that hepatic stem/progenitor cells can drive carcinogenesis of HCC and be a source of tumor initiation is historically well documented. Lee et al.^4^ recently provided evidence to support the crucial role of oval cells (progenitor cells) in restricting cellular tumorigenesis in the liver. This study demonstrated that mammalian Hippo–Salvador pathway restricts the proliferation of hepatic oval cells and thereby controls liver size and prevents the development of oval cell-derived tumors. However, other studies challenge the concept of progenitor cell origin of HCC. Font-Burgada et al.^21^ found that the periportal hepatocytes (progenitor cells) underwent extensive proliferation and replenished liver mass after chronic hepatocyte-depleting injuries but did not give rise to HCC, by using genetic lineage tracing in three distinct HCC models.

Interestingly, mature hepatocytes are also characterized by longevity and remarkable regenerative potential without loss of functional properties^6^, and there are also some studies showing that hepatocarcinogenesis is driven by transformation of hepatocytes. This concept is well supported by several mouse models of hepatocarcinogenesis^22,23^. Studies from Simone Jörs et al.^5^ suggest that the neoplastic lesions in the DEN and Mdr2^−/−^ HCC models are not derived from ductular reactive cells but rather originate from hepatocytes.

Bone marrow derived stem cells, a kind of pluripotent stem cell, have been proven to be involved in liver damage repair and could differentiate into mature hepatic cells and bile duct cells in injured liver^7^. More importantly, bone marrow is also proven as a potential source of hepatic oval cells^24^. Total number of divisions of the normal self-renewing cells is strongly correlated with the risk of cancers^25^. It should be noted that bone marrow-derived cells have strong plasticity^26^ and have been already confirmed to be potential initiating cells of malignant tumors in various types of cancer^10^. However, the actual role of bone marrow derived cells in the process of the initiation and progression of HCC remains largely unknown.

Our results have shown that BMDCs are an important origin of HCC, by using single sequencing in a DEN-induced HCC model in bone marrow transplanted mice. Due to the extremely long experimental period and relatively small tumor size, in our study, the numbers of HCC cells available for sequencing and histopathological analyses were still very limited. Nevertheless, the results from each HCC-bearing mouse indicated a clear role for BMDCs in HCC formation. At this stage, we still lack the data to confirm this observation in human HCC.

However, we speculate that during liver repair, in the process of the differentiation of BMDCs into hepatocytes, these cells are vulnerable to chemical- or inflammation-induced mutagenesis and tumorigenesis. Thus, these results are also consistent with the conclusion from lineage-tracing studies, and provide important clues for HCC prevention and intervention^27^.
