# Supplementary material for: The role of bone marrow-derived cells in the origin of liver cancer revealed by single-cell sequencing
Source: Cancer Biol Med. 2020 Feb 15;17(1):142–53. doi: 10.20892/j.issn.2095-3941.2019.0369 (PMC7142842; doi:10.20892/j.issn.2095-3941.2019.0369)
Supplement: Supplementary file 1 [file cbm-17-142-s001.pdf]

Supplementary materials

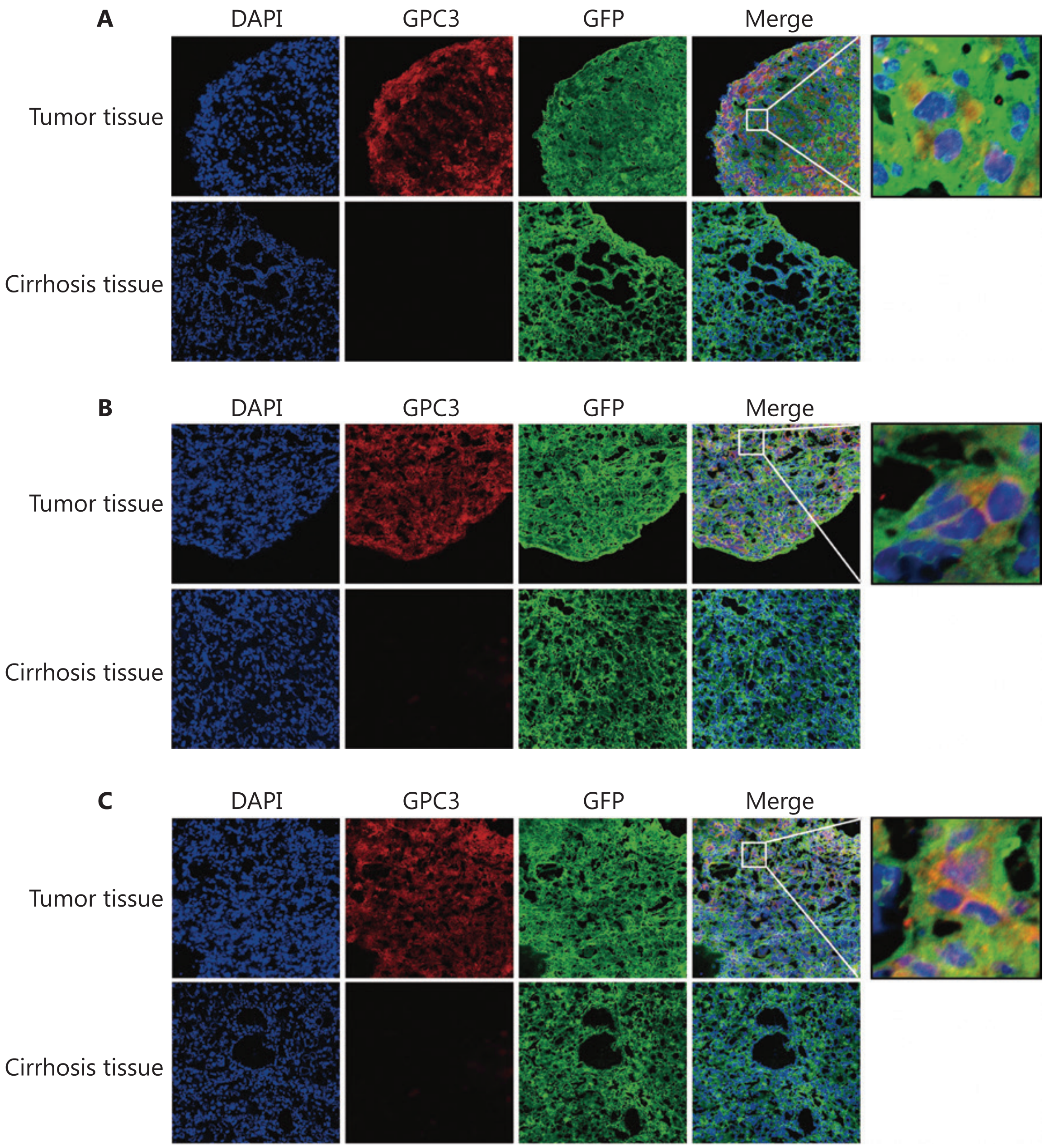

Figure S1 Immunofluorescent colocalization of green fluorescent protein (GFP) and glypican 3 (GPC3) in A. Mouse #14; B. Mouse #22 and C. Mouse #30.

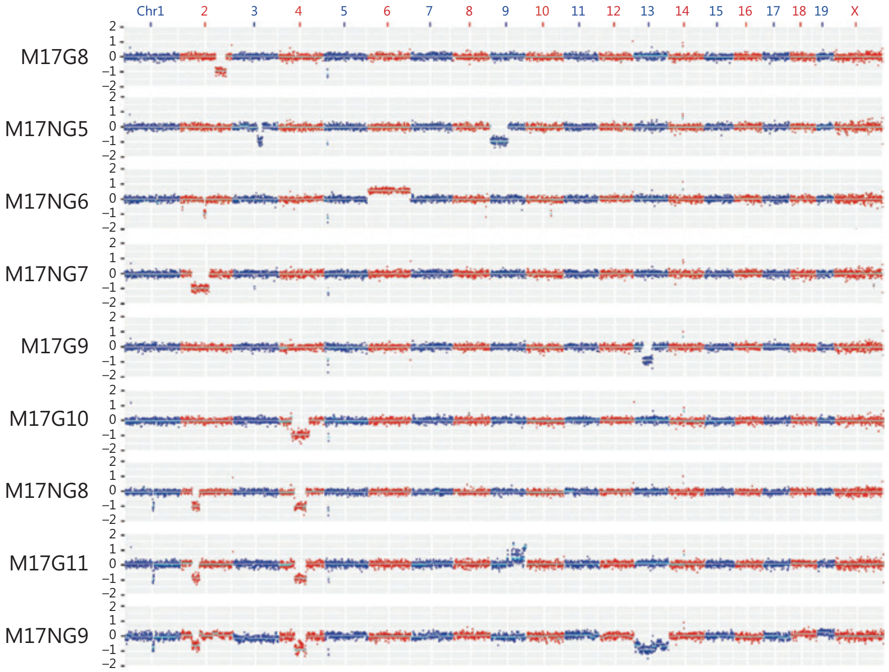

Figure S2 Copy number alteration (CNA) patterns of diethylnitrosamine (DEN)-induced hepatocellular carcinoma (HCC) in Mouse #17. The copy number profile (blue and red dots) indicated by log2 (copy number ratio) is plotted along the genome with a bin size of 500 kb. (G: green cell, NG: non-green cell).

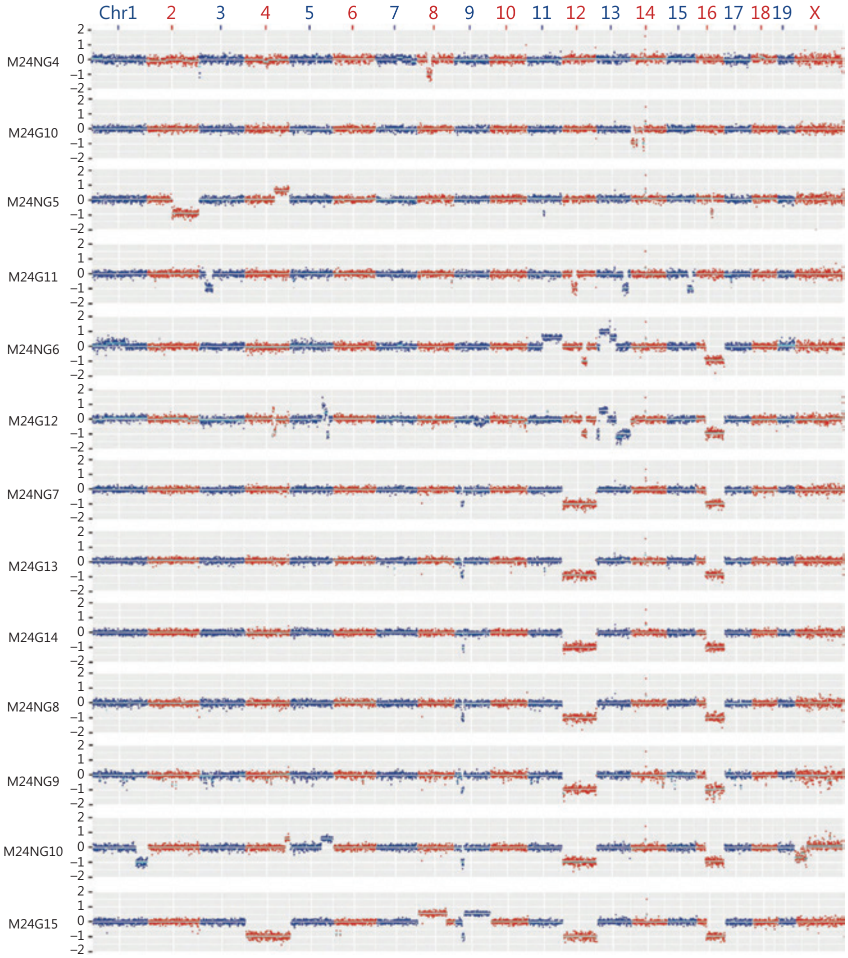

Figure S3 CNA patterns of DEN-induced HCC in Mouse #24. The copy number profile (blue and red dots) indicated by log2 (copy number ratio) is plotted along the genome with a bin size of 500 kb. (G: green cell, NG: non-green cell). Abbreviations are defined in **Figure S2**.

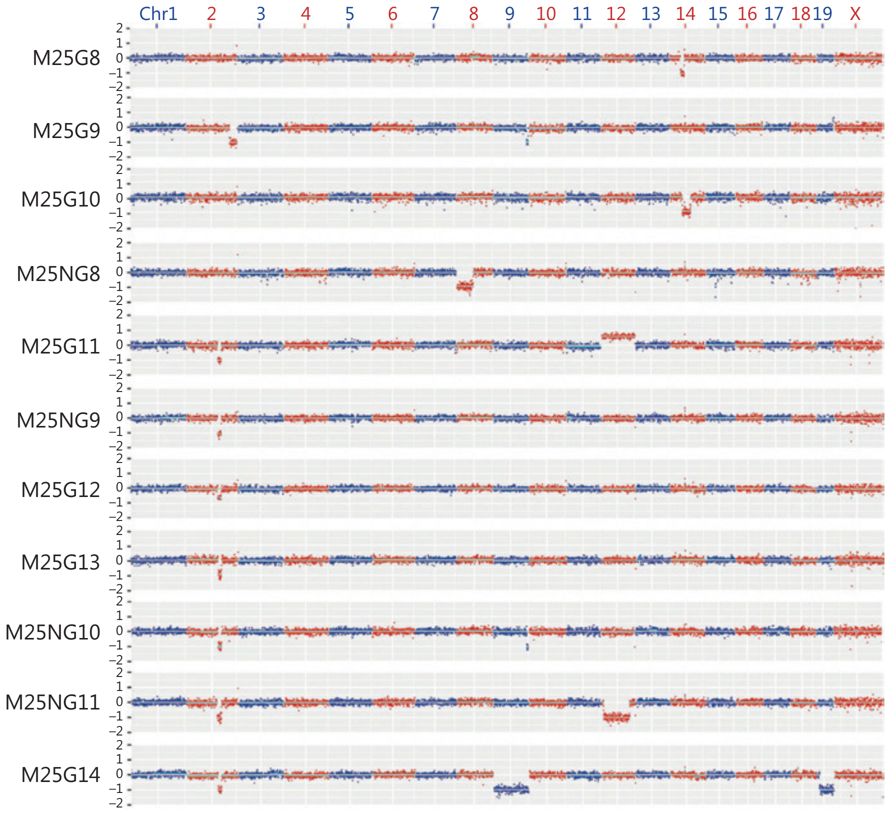

Figure S4 CNA patterns of DEN-induced HCC in Mouse #25. The copy number profile (blue and red dots) indicated by log2 (copy number ratio) is plotted along the genome with a bin size of 500 kb. (G: green cell, NG: non-green cell). Abbreviations are defined in **Figure S2**.

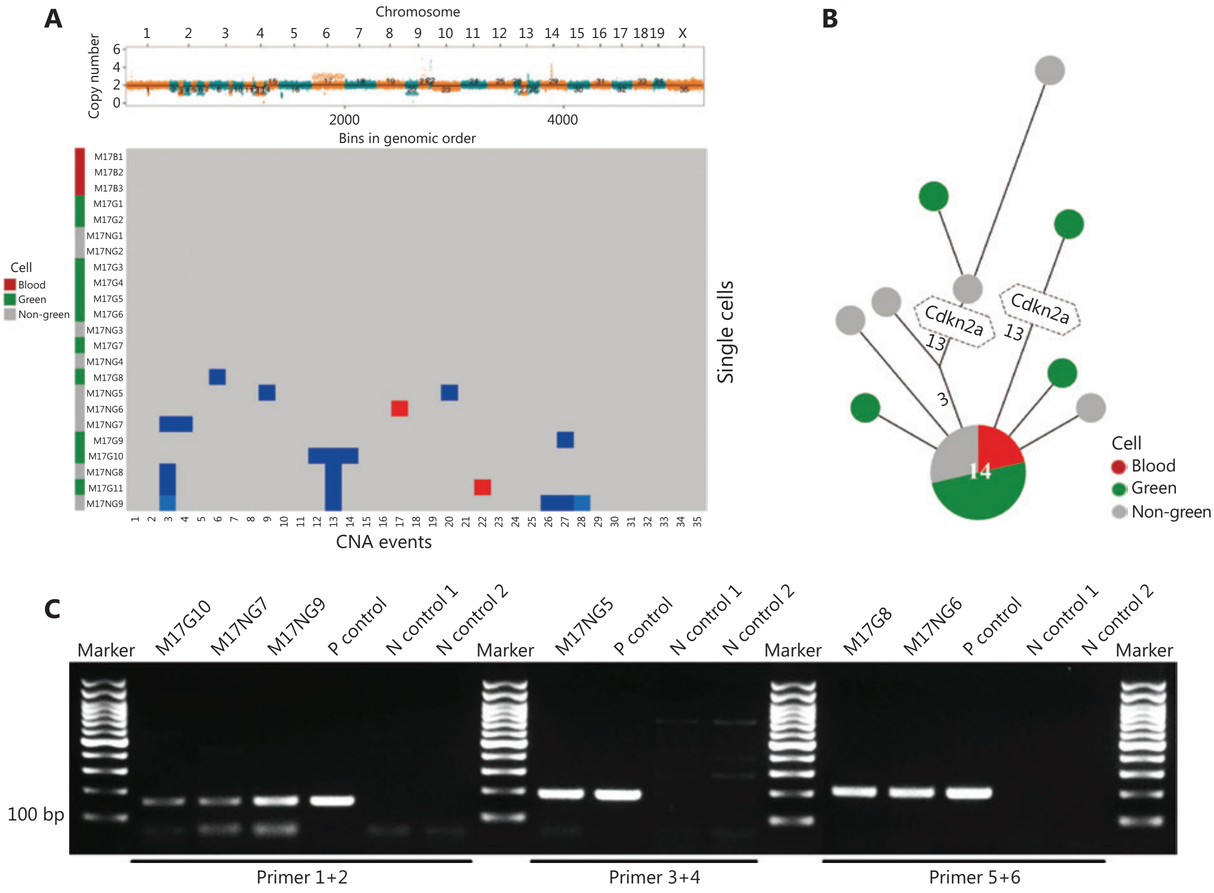

Figure S5 Single-cell CNA revealed the origination and evolution of HCC in Mouse #17. (A) Multiple-cell segmentation (top) and trinary event matrices (bottom) for HCC from whole bone marrow (BM) transplantation mice. (B) A phylogenetic tree was constructed based on the CNA trinary event matrices of single cells. (C) Nested polymerase chain reaction (PCR) was used for detecting green fluorescent protein (GFP) at the single-cell level in Mouse #10 (P control: positive control from the genomic DNA of GFP mouse liver tissue. N control: negative control from wild-type mouse peripheral lymphocytes). Abbreviations are defined in **Figure S2**.

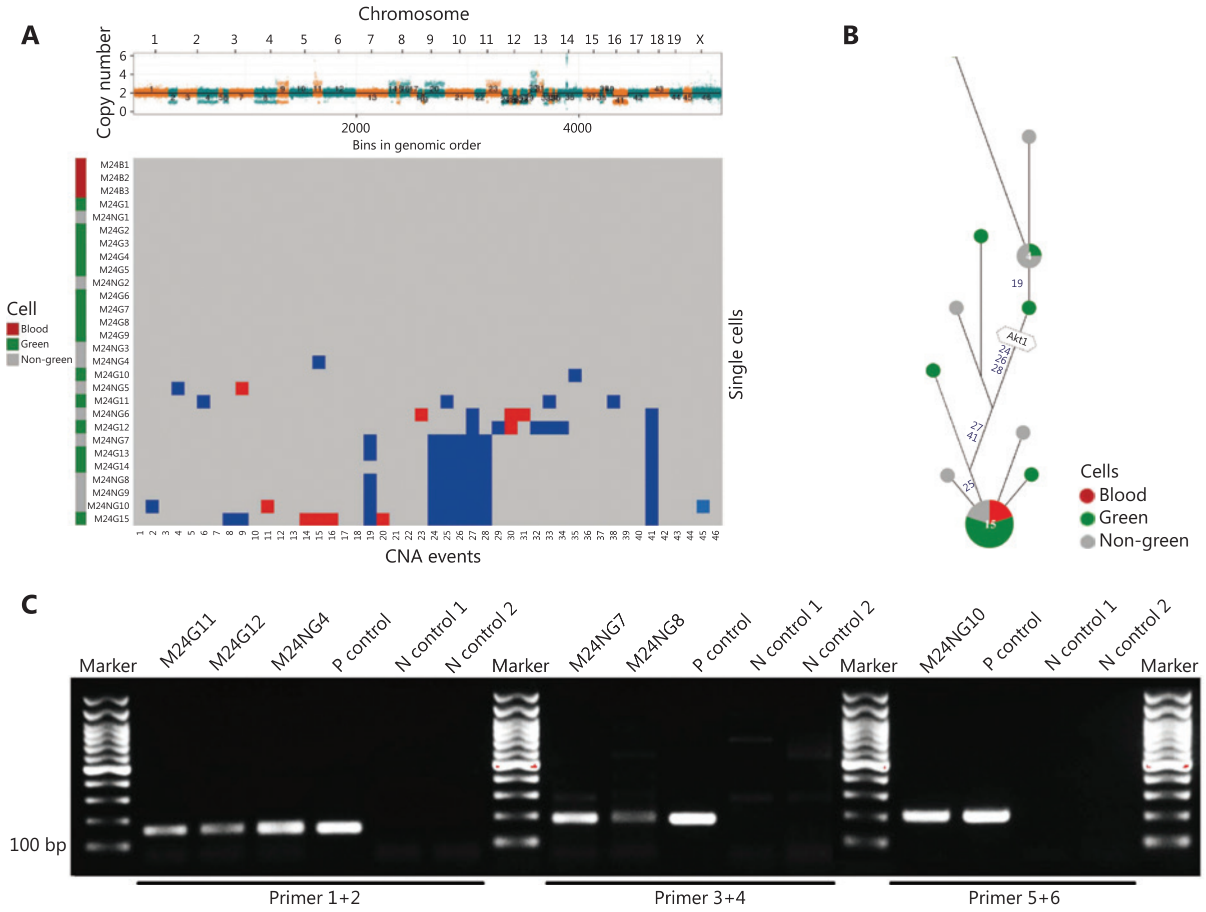

Figure S6 Single-cell CNA revealed the origination and evolution of HCC in Mouse #24. (A) Multiple-cell segmentation (top) and trinary event matrices (bottom) for HCC from whole bone marrow-transplanted mice. (B) A phylogenetic tree was constructed based on the CNA trinary event matrices of single cells. (C) Nested PCR for detecting GFP at the single-cell level in Mouse #25 (P control: positive control from the genomic DNA of GFP mouse liver tissue. N control: negative control from wild-type mouse peripheral lymphocytes). Abbreviations are defined in **Figures S1 and S2**.

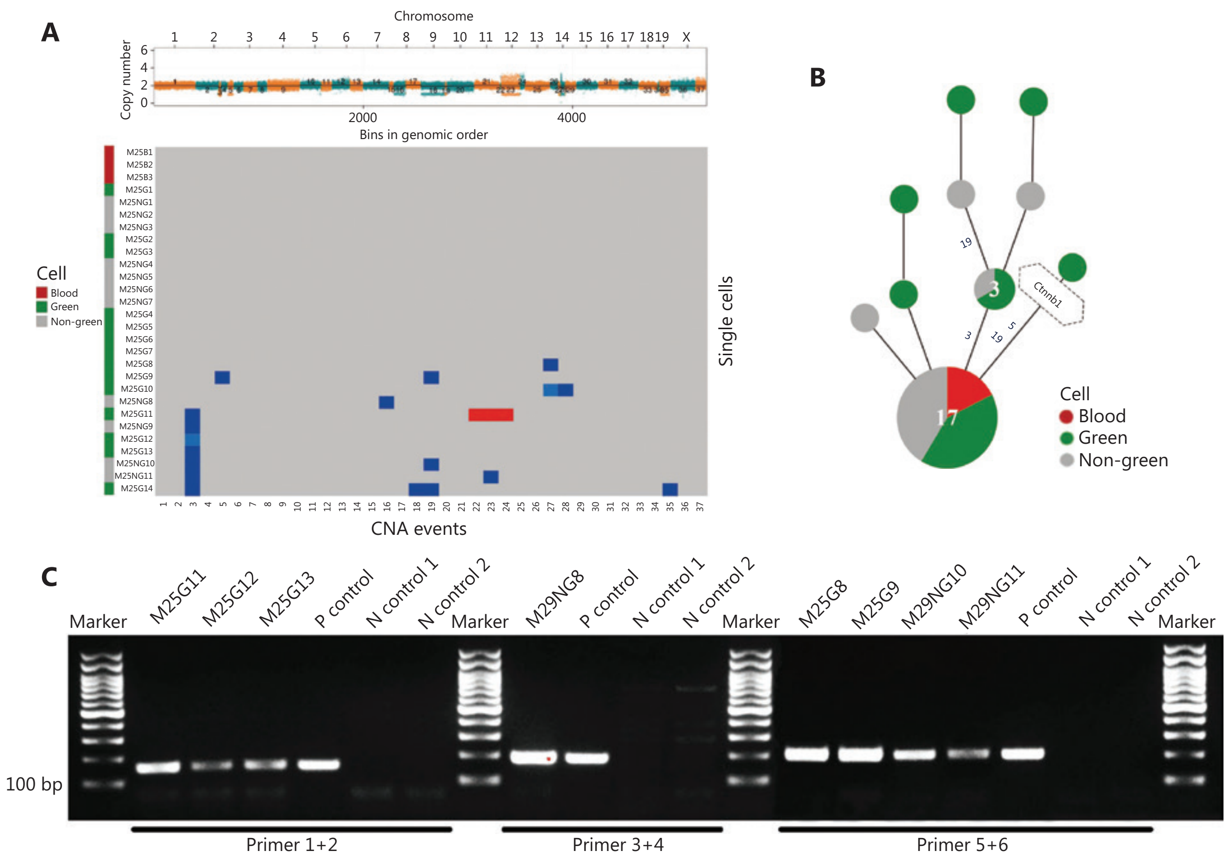

Figure S7 Single-cell CNA revealed the origination and evolution of HCC in Mouse #25. (A) Multiple-cell segmentation (top) and trinary event matrices (bottom) for HCC from whole bone marrow-transplanted mice. (B) A phylogenetic tree was constructed based on the CNA trinary event matrices of single cells. (C) Nested PCR for detecting GFP at the single-cell level in Mouse #29 (P control: positive control from the genomic DNA of GFP mouse liver tissue. N control: negative control from wild-type mouse peripheral lymphocytes). Abbreviations are defined in **Figures S1 and S2**.

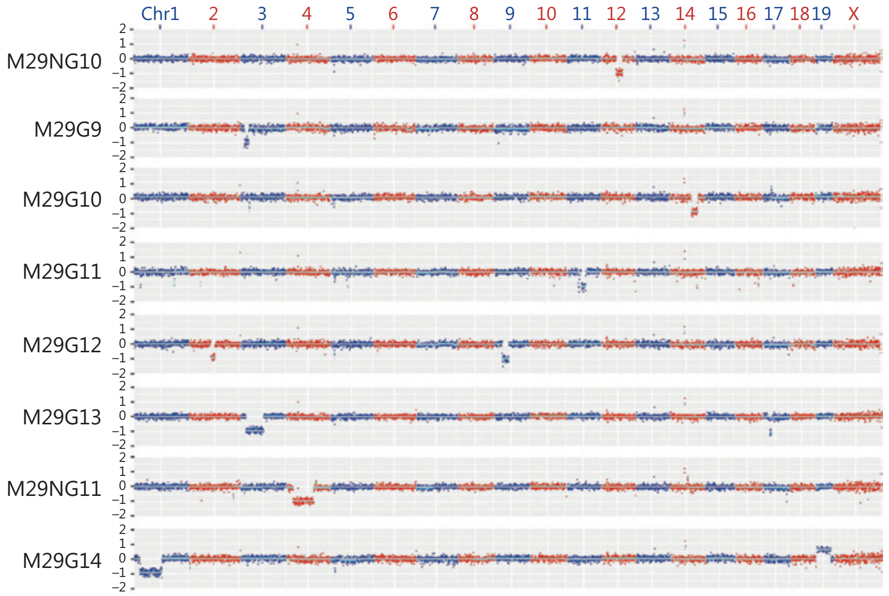

Figure S8 CNA patterns of DEN-induced HCC in Mouse #29. The copy number profile (blue and red dots) indicated by log2 (copy number ratio) is plotted along the genome with a bin size of 500 kb. (G: green cell, NG: non-green cell). Abbreviations are defined in **Figures S1 and S2**.

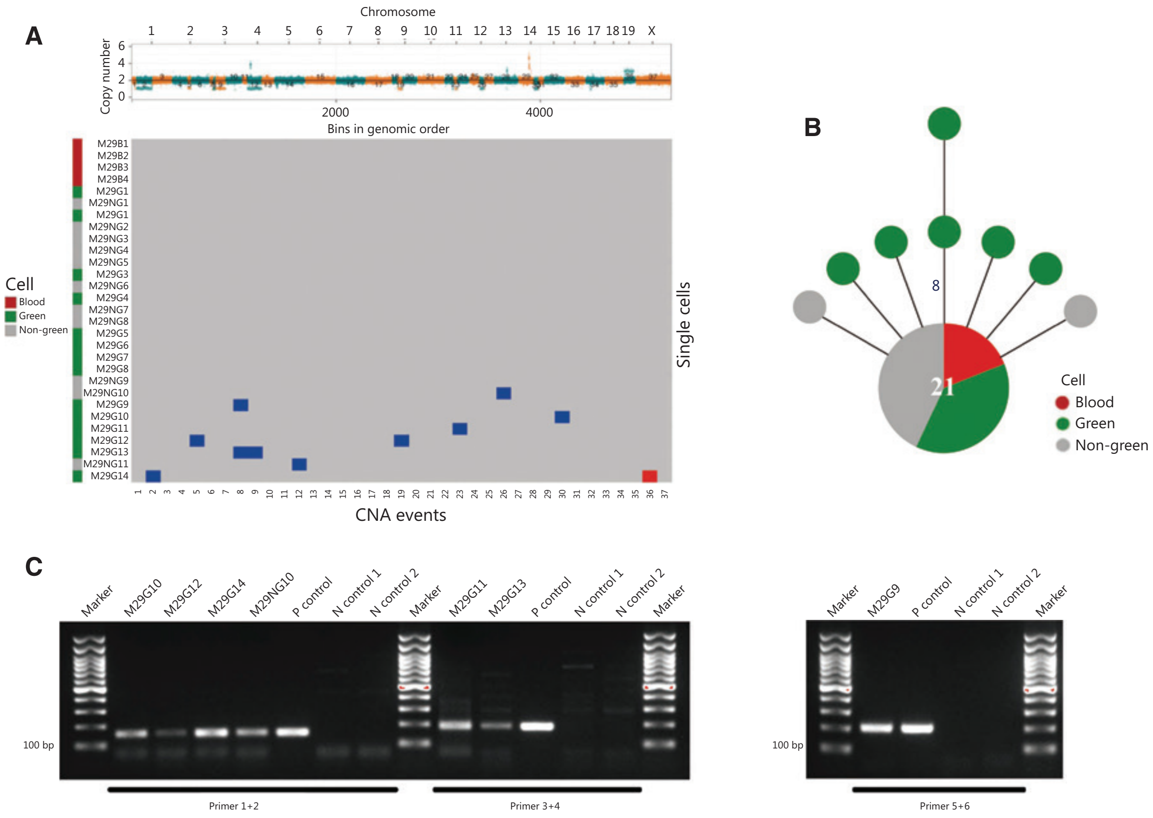

Figure S9 The analysis of an early stage HCC case (Mouse #29) in the whole bone marrow transplantation mice. (A) Multiple-cell segmentation (top) and trinary event matrices (bottom) for HCC in Mouse 24. (B) A phylogenetic tree was constructed based on the CNA trinary event matrices of single cells. (C) Nested PCR for detecting GFP at the single-cell level in Mouse #24. (P control: positive control from the genomic DNA of GFP mouse liver tissue. N control: negative control from wild-type mouse peripheral lymphocytes). Abbreviations are defined in **Figures S1 and S2**.

Table S1 Primers for quantitative polymerase chain reaction to test the genomic integrity of amplification products

Primer	Sequence
1	F-GGAATGGGATTAGGAATGTATCAGC
	R-GCTAATCCATGTGCTAGGCTTACCT
2	F-GTCCCTGAAATTATTGAAAGGGATG
	R-TTCAGGACATCTTCCATCTCATAGC
3	F-GAAGCGAATCACAGTAGAGAACAGC
	R-CCCTTACAGGCTGAGTTCTTCAAAT
4	F-CCCACTGATTTTAACCAGGTTCTCT
	R-GGAATCATTTCTTCTCTGCCAAAGT
5	F-AGCCTACAAGCAGTTTATTCCCAAC
	R-CCTTAAATCGTTCGGAGTTTCTTGT
6	F-TGTTATCAGTAAGGGAGCTGCAGTG
	R-AGCAGAGAACTCCCAGAAAGGTAT
7	F-CCTGACTACTGTCTGGATTCAAGGA
	R-CATCTCCTTTTCCTTCTTGCCTTTA
8	F-GATGCATGAATCAATCTTTGCTACC
	R-GAGCCCTCACAAGTACAATCAGAGA

F, means forward primers; R, means reverse primers.

Table S2 Green fluorescent protein sequence primers

Primer	Sequence
1	F-GGTGAACTTCAAGATCCGCC
	R-CTTGTACAGCTCGTCCATGC
2	F-GGTGAACTTCAAGATCCGCC
	R-TCGTTGGGGTCTTTGCTCAG
3	F-CGTAAACGGCCACAAGTTCAG
	R-GCGGACTTGAAGAAGTCGTG
4	F-CGTAAACGGCCACAAGTTCAG
	R-AAGTCGTGCTGCTTCATGTG
5	F-AGGAGCGCACCATCTTCTTC
	R-CTCGATGTTGTGGCGGATCT
6	F-GACGACGGCAACTACAAGAC
	R-GGCGGATCTTGAAGTTCACC

F, means forward primers; R, means reverse primers.

Table S3 Diethylnitrosamine (DEN)-induced hepatocellular carcinoma (HCC) in the whole bone marrow-transplanted C57BL/6 mice

Mice number	Strain	Tumor	Diameter	Induction time	Application
Mouse #1	C57BL/6	√	–	14 weeks (DIE)	–
Mouse #2	C57BL/6	×	–	16 weeks	–
Mouse #3	C57BL/6	×	–	16 weeks	–
Mouse #4	C57BL/6	×	–	16 weeks	–
Mouse #5	C57BL/6	√	5 mm	16 weeks	Sequencing
Mouse #6	C57BL/6	√	2 mm	16 weeks	IHC, IF (**Figure 1**)

IHC, immunohistochemistry; IF, immunofluorescence.

Table S4 Single-cell GFP PCR statistical results

Number	Tumor cell	Total PCR+	Green cell PCR+	Non-green cell PCR+
Mouse #5	25	17	5/8	12/17
Mouse #10	22	14	5/11	9/11
Mouse #17	9	6	2/4	4/5
Mouse #24	13	6	2/6	4/7
Mouse #25	11	8	5/7	3/4
Mouse #29	8	7	6/6	1/2

PCR, polymerase chain reaction; GFP, green fluorescent protein.

Table S5 DEN-induced HCC in the following validation group

Mice number	Strain	Tumor	Diameter	Induction time	Application
Mouse #7	C57BL/6	×	–	16 weeks	–
Mouse #8	C57BL/6	×	–	12 weeks (died)	–
Mouse #9	C57BL/6	×	–	16 weeks	–
Mouse #10	C57BL/6	√	5.5 mm	16 weeks	Sequencing
Mouse #11	C57BL/6	×	–	16 weeks	–
Mouse #12	C57BL/6	×	–	16 weeks	–
Mouse #13	C57BL/6	×	–	16 weeks	–
Mouse #14	C57BL/6	√	2.5 mm	16 weeks	IF (**Figure 3 and S1**)
Mouse #15	C57BL/6	×	–	16 weeks	–
Mouse #16	C57BL/6	×	–	16 weeks	–
Mouse #17	C57BL/6	√	5 mm	16 weeks	Sequencing
Mouse #18	C57BL/6	×	–	16 weeks	–
Mouse #19	C57BL/6	×	–	16 weeks	–
Mouse #20	C57BL/6	×	–	16 weeks	–
Mouse #21	C57BL/6	×	–	16 weeks	–
Mouse #22	C57BL/6	√	2 mm	16 weeks	IF (**Figure S1**)
Mouse #23	C57BL/6	×	–	16 weeks	–
Mouse #24	C57BL/6	√	5 mm	16 weeks	Sequencing
Mouse #25	C57BL/6	√	4 mm	16 weeks	Sequencing
Mouse #26	C57BL/6	×	–	16 weeks	–
Mouse #27	C57BL/6	×	–	16 weeks	–
Mouse #28	C57BL/6	×	–	16 weeks	–
Mouse #29	C57BL/6	√	2.5 mm	16 weeks	Sequencing
Mouse #30	C57BL/6	√	2 mm	16 weeks	IF (**Figure 3 and Table S3**)
Mouse #31	C57BL/6	×	–	16 weeks	–

IF, immunofluorescence; HCC, hepatocellular carcinoma; DEN, diethylnitrosamine; √, means tumor formation; ×, means non tumor formation.

Table S6 Important genes associated with human HCC in 6 mice

Chromosome	Position	Mice	Gene
chr1	155500000-195500000	Mouse #5, #10, #24	*Tert*
chr2	126500000-155500000	Mouse #5, #17, #24	*Atxn1*
chr3	23500000-48500000	Mouse #5, #10, #24, #29	*Nras*
chr9	29500000-53500000	Mouse #5, #10, #17, #24, #25, #29	*Cb1, Cyp46a1*
	64500000-115500000	Mouse #5, #10, #24, #25	*Cyp8b1, Dkk1*
chr11	54000000-67000000	Mouse #10, #24, #29	*Ctnnb1*
chr12	3500000-54000000	Mouse #5, #10, #24, #25	*Arid1a, Pik3ca*
	77000000-120500000	Mouse #5, #10, #24, #25	*Cdkn2a*

Chr, chromosome.
